# Gestational age at birth and academic attainment in primary and secondary school in England: Evidence from a national cohort study

**DOI:** 10.1371/journal.pone.0271952

**Published:** 2022-08-17

**Authors:** Neora Alterman, Samantha Johnson, Claire Carson, Stavros Petrou, Jennifer J. Kurinzcuk, Alison Macfarlane, Elaine Boyle, Maria A. Quigley

**Affiliations:** 1 National Perinatal Epidemiology Unit (NPEU), Nuffield Department of Population Health, University of Oxford, Oxford, United Kingdom; 2 Department of Health Sciences, University of Leicester, Leicester, United Kingdom; 3 Nuffield Department of Primary Care Health Sciences, University of Oxford, Oxford, United Kingdom; 4 Department of Health Sciences, City University, London, United Kingdom; Western University, CANADA

## Abstract

Preterm birth (<37 weeks’ gestation) is a risk factor for poor educational outcomes. A dose-response effect of earlier gestational age at birth on poor primary school attainment has been observed, but evidence for secondary school attainment is limited and focused predominantly on the very preterm (<32 weeks) population. We examined the association between gestational age at birth and academic attainment at the end of primary and secondary schooling in England. Data for children born in England from 2000–2001 were drawn from the population-based UK Millennium Cohort Study. Information about the child’s birth, sociodemographic factors and health was collected from parents. Attainment on national tests at the end of primary (age 11) and secondary school (age 16) was derived from linked education records. Data on attainment in primary school was available for 6,950 pupils and that of secondary school was available for 7,131 pupils. Adjusted relative risks (aRRs) for these outcomes were estimated at each stage separately using modified Poisson regression. At the end of primary school, 17.7% of children had not achieved the expected level in both English and Mathematics and this proportion increased with increasing prematurity. Compared to full term (39–41 weeks) children, the strongest associations were among children born moderately (32–33 weeks; aRR = 2.13 (95% CI 1.44–3.13)) and very preterm (aRR = 2.06 (95% CI 1.46–2.92)). Children born late preterm (34–36 weeks) and early term (37–38 weeks) were also at higher risk with aRR = 1.18 (95% CI 0.94–1.49) and aRR = 1.21 (95% CI 1.05–1.38), respectively. At the end of secondary school, 45.2% had not passed at least five General Certificate of Secondary Education examinations including English and Mathematics. Following adjustment, only children born very preterm were at significantly higher risk (aRR = 1.26 (95% CI 1.03–1.54)). All children born before full term are at risk of poorer attainment during primary school compared with term-born children, but only children born very preterm remain at risk at the end of secondary schooling. Children born very preterm may require additional educational support throughout compulsory schooling.

## Introduction

Preterm birth (before 37 completed weeks of gestation) accounts for approximately 11% of births globally [1]. In England, in the early 2000s, just over 7% of children were born preterm [2]. The fetal brain develops considerably during the third trimester of pregnancy, when cortical volume increases four-fold [3]. Infants born prematurely must mature in an atypical extra-uterine environment, leading to alterations in brain development, with earlier birth being more disruptive to normal fetal maturation [4]. Children born preterm are therefore at risk of neurodevelopmental sequelae including low IQ, deficits in attention and executive functions, poor visuospatial processing, gross and fine motor deficits, and social-emotional problems [4, 5]. Each of these difficulties may adversely impact the child’s performance at school.

Using a sample representative of the UK, we have previously shown that children born at any gestational age earlier than full term (39–41 weeks) have poorer academic attainment at ages 5 and 7 and are more likely to have special educational needs (SEN) at age 11 when they finish primary school [5–7]. A number of longitudinal studies have shown that cognitive impairments and attention, emotional, and social problems in early childhood remain elevated into adulthood amongst children born very (<32 weeks of gestation) or extremely (<28 weeks of gestation) preterm [8–10]. It might thus be expected that deficits in academic attainment among children born very preterm would persist throughout schooling. However, few studies have followed children born preterm up until the end of compulsory education [8, 11, 12] and fewer have examined these long-term outcomes in relation to the full spectrum of gestational age at birth [13–15].

Evidence from a birth cohort from England suggests that, as a group, children born preterm have poorer academic performance, on average, than term-born children during compulsory education, but they experience some catch-up in academic attainment compared with their term-born peers [14]. An additional study has shown that at the end of secondary school, preterm birth may no longer be a risk factor for poor attainment when other factors are accounted for [15]. This evidence, however, pertains to the broad group of all children born preterm, before 37 weeks of gestation. In contrast, the higher risk sub-group of children born very preterm has been shown to have lower academic attainment throughout primary school [15–17] and on achievement tests in secondary school [11, 18, 19]. Cross-sectional studies carried out at the end of compulsory education in other high-income countries have also described a dose-response effect of earlier gestational age at birth on the likelihood of not completing basic schooling or failing final examinations [13, 20].

The cognitive deficits associated with preterm birth may impact differently on different school subjects. In particular, it is well documented that the poorer performance of children born very preterm is more pronounced in mathematics than in other school subject [21, 22]. However, no studies have separately assessed attainment by subject across the age range of compulsory schooling in relation to gestational age at birth.

The possible neurodevelopmental outcomes of preterm birth, including an increased risk for cognitive, motor, attention and social-emotional problems, may adversely affect various facets of learning. We hypothesized that birth at earlier gestational ages would be independently associated with poorer educational attainment. Our aim was therefore to examine the association between gestational age at birth across the full gestational age spectrum and attainment at two important stages of schooling in England–at the end of primary school (age 11) and at the end of secondary school (age 16).

## Methods

### Millennium cohort study

The Millennium Cohort Study (MCS) is a nationally representative prospective cohort study of 18,818 children born in the UK, including 11,695 children born in England from 1^st^ September 2000 to 31^st^ August 2001 who entered school in the same academic year and on which the current study is focused. The universal Child Benefit register was used for sampling children at 9–10 months of age. The MCS therefore does not include children that died prior to this age, but these constituted only 0.55% of births [23]. The MCS has a cluster-stratified design with oversampling of clusters with a high proportion of disadvantaged families or ethnic minorities to ensure adequate representation of such families [24]. Families were interviewed at home; the first interview, usually with the mother conducted when the child was aged 9–10 months [25, 26]. Information about the birth as well as health and demographic factors was collected at this time. When the children reached seven years of age, families were asked for consent to link study data to their child’s education records [27]. Data for 93.3% of the children living in England at this age were linked to their records held in the Department for Education’s National Pupil Database (NPD) [27–29].

### Gestational age at birth

Gestational age at birth in weeks was ascertained from the mother’s report of expected date of delivery and actual date of birth. This MCS variable was previously compared with information from linked hospital birth records and found to be valid, except for births post term (42 weeks or more) [30]. Completed gestational weeks were categorised as 23–31 weeks (very preterm), 32–33 weeks (moderately preterm), 34–36 weeks (late preterm), 37–38 weeks (early term), 39–41 weeks (full term) (Table 1) [31].

**Table 1:** Characteristics of the children included in the Key Stage 4 (KS4; secondary school, age 16) analysis study sample.

	Very preterm	Moderately preterm	Late preterm	Early term	Full term	Total
	<32 weeks	32–33 weeks	34–36 weeks	7–38 weeks	39–41 weeks	N (%)
	N (%) ^1^	N (%)	N (%)	N (%)	N (%)	
	81 (1.1)	80 (1.1)	408 (5.8)	1,498 (21.3)	5,064 (70.7)	7,131 (100.0)
***Sociodemographic characteristics***						
**Maternal age, mean ^1^ (SD)**	29.1 (5.52)	28.8 (6.21)	28.8 (6.37)	28.9 (5.86)	28.5 (5.87)	28.6 (5.90)
**White child ethnicity**	56 (76.5)	62 (88.0)	326 (85.5)	1,119 (81.1)	3,974 (85.2)	5,537 (84.3)
**Partnership status**						
Married	49 (54.6)	49 (63.2)	241 (55.1)	986 (61.7)	3,171 (59.0)	4,496 (59.3)
Cohabiting	20 (27.8)	- ^**2**^	111 (29.2)	325 (24.5)	1,262 (27.1)	1,742 (26.7)
Single mother	12 (17.6)	-	56 (15.8)	187 (13.8)	631 (14.0)	893 (14.0)
**Maternal education**						
Higher (University)	28 (31.6)	25 (29.9)	120 (27.3)	436 (27.5)	1,638 (31.5)	2,247 (30.4)
Medium (Advanced-level)	-	-	49 (11.5)	205 (13.2)	686 (13.8)	954 (13.4)
Lower (GCSE)	29 (36.9)	35 (46.7)	183 (45.7)	582 (41.1)	1,901 (39.5)	2,730 (40.2)
Overseas/other or none	-	-	56 (15.6)	275 (18.2)	839 (15.3)	1,200 (16.0)
**Household socioeconomic class**						
Managerial/professional	21 (25.9)	33 (45.3)	179 (42.5)	643 (42.9)	2,260 (45.0)	3,136 (44.2)
Intermediate	25 (29.6)	19 (23.8)	70 (17.7)	298 (19.9)	994 (19.7)	1,406 (19.8)
Routine/manual or unemployed	35 (44.5)	28 (31.0)	159 (39.8)	557 (37.2)	1,810 (35.3)	2,589 (36.0)
***Pregnancy and postnatal characteristics***						
**Male**	39 (52.1)	46 (63.6)	210 (51.4)	776 (52.5)	2,507 (49.8)	3,578 (50.6)
**Birthweight in Kg, mean ^1^ (SD)**	1.23 (0.39)	1.95 (0.42)	2.63 (0.51)	3.14 (0.49)	3.49 (0.56)	3.33 (0.59)
**Multiple birth**	19 (22.5)	15 (20.6)	60 (15.0)	68 (4.4)	18 (0.3)	180 (2.5)
**Firstborn child**	35 (43.4)	45 (58.4)	175 (41.6)	543 (37.1)	2,097 (41.9)	2,895 (41.0)
**Maternal smoking**						
No	46 (56.0)	47 (57.8)	266 (63.0)	1,054 (65.6)	3,568 (66.7)	4,981 (66.0)
Gave-up in pregnancy	11 (11.2)	19 (25.0)	48 (12.0)	147 (11.0)	591 (12.9)	816 (12.6)
Smoked in pregnancy	24 (32.7)	14 (17.3)	94 (25.0)	297 (23.4)	905 (20.4)	1,334 (21.4)
**Labour induced**	-	18 (17.3)	107 (26.0)	411 (27.2)	1,361 (27.0)	1,906 (26.7)
**Mode of birth**						
Vaginal delivery	31 (41.9)	34 (37.3)	264 (65.4)	1,001 (67.4)	4,274 (85.2)	5,604 (79.3)
Planned caesarean	-	11 (13.0)	39 (10.1)	335 (22.7)	263 (5.0)	655 (9.1)
Emergency caesarean	-	35 (49.7)	105 (24.5)	161 (9.9)	516 (9.8)	859 (11.6)
**Ever breastfed**	54 (67.7)	62 (74.5)	284 (64.9)	1,107 (69.3)	3,827 (71.9)	5,334 (71.0)
**Month of birth**						
September-December	20 (22.0)	30 (34.5)	136 (33.2)	535 (36.3)	1,754 (34.6)	2,475 (34.8)
January-April	27 (33.6)	21 (27.3)	147 (34.6)	512 (33.7)	1,590 (31.0)	2,297 (31.8)
May-August	34 (44.4)	29 (38.2)	125 (32.2)	451 (30.0)	1,720 (34.4)	2,359 (33.5)

^1^ Percentages and means are weighted to account for study design, non-response to the MCS and non-response at age seven

^2^ Some cells with small values have been supressed to avoid risk of disclosure

### Academic attainment

In England, the national curriculum is divided into four ‘Key Stages’ spanning the full period of compulsory education: Key Stage 1 (KS1; ages 5–7); Key Stage 2 (KS2; ages 7–11); Key Stage 3 (KS3; ages 11–14) and Key Stage 4 (KS4; ages 14–16) [32]. All state-funded schools and some independent schools follow this curriculum and report results to the NPD [33].

At the end of primary school, when children are 11 years of age, pupils’ attainment at KS2 is assessed using standardised tests in English and Mathematics, alongside a teacher’s assessment of their schoolwork in these subjects. Our study’s main outcome measure for KS2 was not achieving the expected level of attainment (Level 4 or above) in both English and Mathematics [34]. Additionally, we examined the association between gestational age at birth and not achieving the expected level of attainment (Level 4 or above) in English and in Mathematics separately. Table 2 details the expected attainment levels at KS2.

**Table 2:** Comparison between educational outcome measures in the Millennium Cohort Study samples and published national statistics at Key Stages 2 (age 11) and 4 (age 16).

Outcome measure	Expected level or passing grade	% Achieving measure in MCS study sample (95% CI)	% Achieving measure in England ^1^
***Primary school—Key Stage 2 (year 2012)***			
Achieved expected level in English and in Mathematics ^2^ (**main outcome**)	Level 4 or above [34]	82.3 (81.1, 83.5)	79 [34]
Achieved expected level in English	Level 4 or above [34]	87.7 (86.6, 88.7)	85 [34]
Achieved expected level in Mathematics	Level 4 or above [34]	86.4 (85.4, 87.4)	84 [34]
***Secondary school—Key Stage 4 (year 2017)***			
Achieved expected outcome in GCSEs (**main outcome**)	At least 5 GCSEs at grade A*-C including English and Mathematics [35]	54.8 (52.9, 56.7)	53.5 in 2016 ^3^ [35]
Achieved pass in English element ^4^	Grade 9–4 [36]	72.2 (70.5, 73.8)	75.5 (state-funded schools) [36]
Achieved pass in Mathematics element ^5^	Grade 9–4 [36]	67.0 (65.3, 68.7)	69.2 (state-funded schools) [36]
		**Mean measure in MCS study sample (95% CI)**	**Mean measure in England**
Attainment 8 ^5^mean score	-	45.5 (44.8, 46.3)	44.6 [36]

^1^ Note that the MCS is based on a birth cohort of children born in England whereas the published Department for Education statistics include all children who were at school in England and had those assessments.

^2^ In the year 2012 when the MCS children finished KS2, the level of attainment in English was measured by a standardized test in reading and a teacher’s assessment in writing. The level of attainment in Mathematics was measured by standardized tests.

^3^ We compared MCS results to statistics published in 2016, as this was the final year when this statistic was published in annual reports of the Department for Education.

^4^ Passing grade for the English element of the ‘English Baccalaureate’ is defined as grade 4 (equivalent to C in previous scoring system) or above in either English language or English literature GCSEs (or A*-C grades in approved AS levels (Advanced Subsidiary, i.e. the first year of A levels), with entries into both language and literature [36]

^4^ Passing grade for the Mathematics element of the ‘English Baccalaureate’ is defined as grade 4 (equivalent to C in previous scoring system) or above in Mathematics GCSE or A*-C in approved AS levels (Advanced Subsidiary, i.e. the first year of Advanced-levels) [36]

^5^ Achievement in up to 8 qualifications including English (double-weighted if both language and literature are taken), Mathematics (double weighted), 3 further qualifications that count in ‘English Baccalaureate’ and 3 further qualifications that can be GCSE qualifications (including ‘English Baccalaureate’ subjects) or any other non-GCSE qualification [36].

At the end of secondary school, when children are 16 years of age, the majority of pupils take General Certificate of Secondary Education (GCSE) examinations in English, Mathematics and additional subjects (most often nine in total). Our main outcome measure for this stage (KS4) was not achieving at least five GCSE passes at Grade A*-C (or 9–4 if based on the reformed scoring system) [33], including in English and Mathematics (defined in Table 2). This outcome was used by the Department for Education as an annual headline statistic until 2016 [35], and was a common pre-requisite for Advanced-level studies, which are subsequently required for admission to higher education. We also examined separately whether pupils had not achieved a passing grade in English or in Mathematics GCSE [36]. In addition, we analysed pupils’ ‘Attainment 8’ score, which is a combined score of attainment in up to eight subjects; these include English and Mathematics (double weighted) and six further subjects, some of which may be technical or vocational awards (Table 2) [36].

### Exclusions and missing data

**Fig 1:**
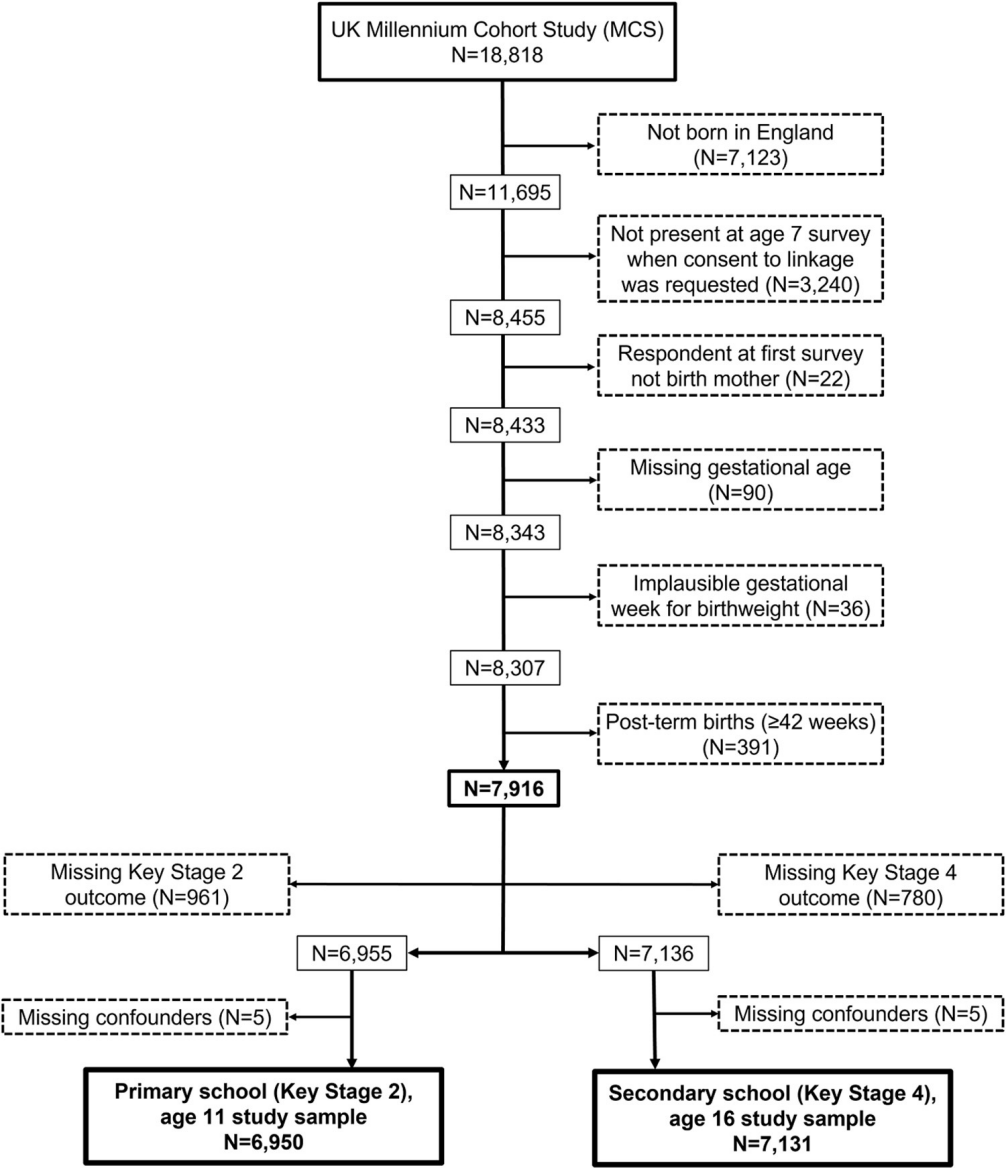
Millennium cohort study children included in the present study, born 2000–2001 in England.

Of the MCS children born in England and recruited to the study, 72.3% participated in the age seven survey when consent to linkage with the NPD was sought (Fig 1). Children were eligible for inclusion if the main respondent to the initial survey was the birth mother and if gestational age was non-missing and plausible given the reported birthweight [37]. Post-term births were not eligible due to lower quality of data on gestational age [30].

Of the 7,916 children eligible for inclusion in the study, we excluded children with missing educational data for the analyses relevant to each assessment age as well as missing data on confounders. This resulted in 6,950 and 7,131 children being included in the analysis for primary and secondary school attainment respectively (Fig 1), with 94% of children being included in both analyses (Table 3, bottom). Educational data for primary school attainment were missing for 961 (12.1%) of all pupils eligible to be included in the study and this proportion was 15.2%, 21.1%, 12.9% and 11.4% for the pupils born very preterm, moderately preterm, late preterm, and early term, respectively. Educational data for secondary school attainment were missing for 780 (9.7%) of eligible pupils (Table 3), and this proportion was higher among children born moderately and late preterm (19.5% and 14.4% respectively).

**Table 3:** Proportion of missing Key Stage 4 (KS4; secondary school; age 16) data within the Millennium Cohort Study sample.

	Non-missing KS4 data	Missing KS4 data	Total
	N (%) ^1^	N (%)	N (%)
**Total**	7,136 (90.3) ^2^	780 (9.7)	7,916 (100)
**Gestational age**			
Very preterm <32 weeks	81 (93.6)	8 (6.4)	89 (100)
Moderately preterm 32–33 weeks	82 (80.5)	18 (19.5)	100 (100)
Late preterm 34–36 weeks	408 (85.6)	66 (14.4)	474 (100)
Early term 37–38 weeks	1,498 (91.6)	144 (8.4)	1,642 (100)
Full term 39–41 weeks	5,067 (90.4)	544 (9.6)	5,611 (100)
**Maternal education**			
Higher (University)	2,247 (89.*5*)	259 (10.5)	2,506 (100)
Medium (Advanced-level)	954 (89.9)	109 (10.1)	1,063 (100)
Lower (GCSE)	2,730 (91.7)	244 (8.3)	2,974 (100)
Overseas /other or none	1,201 (88.5)	167 (11.5)	1,368 (100)
**Ethnicity**			
White	5,539 (91.1)	523 (8.9)	6,062 (100)
Non-white	1,579 (86.1)	254 (13.9)	1,833 (100)
**Household social class**			
Managerial/professional	3,136 (90.0)	345 (10.0)	3,481 (100)
Intermediate	1,407 (92.3)	123 (7.7)	1,530 (100)
Routine/manual or unemployed	2,593 (89.5)	312 (10.5)	2,905 (100)
**Whether firstborn child**			
Firstborn child	2,896 (89.4)	339 (10.6)	3,235 (100)
Not firstborn	4,240 (90.9)	441 (9.1)	4,681 (100)
**Longstanding illness reported by parent at age seven**			
Longstanding illness	1,339 (90.6)	135 (9.4)	1,474 (100)
No longstanding illness	5,779 (90.5)	623 (9.5)	6,402 (100)
**Neonatal special care**			
Neonatal special care	634 (88.6)	75 (11.4)	709 (100)
No neonatal special care	6,502 (90.5)	705 (9.5)	7,207 (100)
**Month of birth**			
September-December	2,476 (90.5)	275 (9.5)	2,751 (100)
January-April	2,298 (89.6)	255 (10.4)	2,553 (100)
May-August	2,362 (90.8)	250 (9.2)	2,612 (100)
**Key Stage 2 data**			
Non-missing	6,821 (98.0)	138 (2.0)	6,959 (100)
Missing	315 (34.8)	642 (65.2)	957 (100)

^1^ Percentages are weighted to account for sampling design, non-response to the MCS and non-response to the age seven survey

^2^ Five pupils included in this table had missing data on confounders and were not included in the age 16 study sample

### Statistical analyses

We estimated relative risks for the association between gestational age and the relevant educational outcomes at each assessment age separately. Modified Poisson regression was used, since outcomes were common and odds ratios would overestimate relative risks [38]. ‘Full term’ (39–41 weeks) was defined as the reference group. Adjustments were made for a priori factors known to confound the association between gestational age and educational outcomes or to be strongly associated with educational outcomes: mother’s education level (using the National Vocational Qualifications system [39]), multiple birth, child sex and month of birth (i.e. age in school year) [5, 6]. Additional potential confounders included mother’s age, partnership status, household’s highest socio-economic status (based on the National Statistics Socio-economic classification), smoking during pregnancy, ethnicity of the child (white/non-white) and whether the child was the firstborn. The additional potential confounders found to be associated with both the exposure and the main outcome at P-value <0.1 were assessed in separate socio-demographic and perinatal models and then in a combined model, and retained if independently associated with the outcome at P-value <0.05. Models of additional outcomes were adjusted for the same covariates to aid comparability.

For the analysis of ‘Attainment 8’, a continuous score, we used ‘Tobit censored regression’ [40] to calculate the difference in mean score between each gestational age group and the full term reference group. We censored the observations of 147 pupils (2.2% of the sample) that scored zero, likely due to not taking exams, and 23 observations with the maximal score.

All analyses were conducted in Stata MP 16. Analyses accounted for the clustered, stratified design of the MCS using the survey commands, while allowing for within-family clustering for multiples. We used weights provided with the MCS data to account for non-response at the initial survey and attrition of participation at the age seven survey [41].

### Ethical approval

Ethical approval for the initial MCS survey was granted from the South West Multicentre Research Ethics Committee (MREC/01/6/19). Consent to link educational records up to age sixteen was sought in writing from parents or guardians in person during the MCS age seven survey. Ethical approval for the age seven survey was granted from the Yorkshire MREC (07/MRE03/32) [42]. No further approvals were required for this study.

## Results

### Descriptive results

There were 6,950 pupils included in the analyses for primary school attainment and 7,131 pupils included in the analyses for secondary school attainment. Of the pupils with available educational data at either time point, only 6% differed between the two samples (Table 3, bottom), and the characteristics of the two samples were therefore similar. As such, detailed descriptive results are presented for the secondary school sample only, which is the larger of the two (Table 1). Birth at earlier gestational age was associated (P-value <0.1) with lower birthweight and a higher proportion of multiple births in a dose-response pattern. Gestational age was also associated with the potential confounders of maternal age, education level, smoking during pregnancy, child sex, ethnicity, being firstborn and month of birth.

The educational outcomes assessed in our study are detailed in Table 2, alongside published estimates of these outcomes for all pupils in England from the relevant years. The overall proportion or mean score of pupils achieving each outcome in our sample was similar to that seen in national statistics.

### Gestational age and academic attainment at the end of primary school (KS2; age 11)

**Fig 2:**
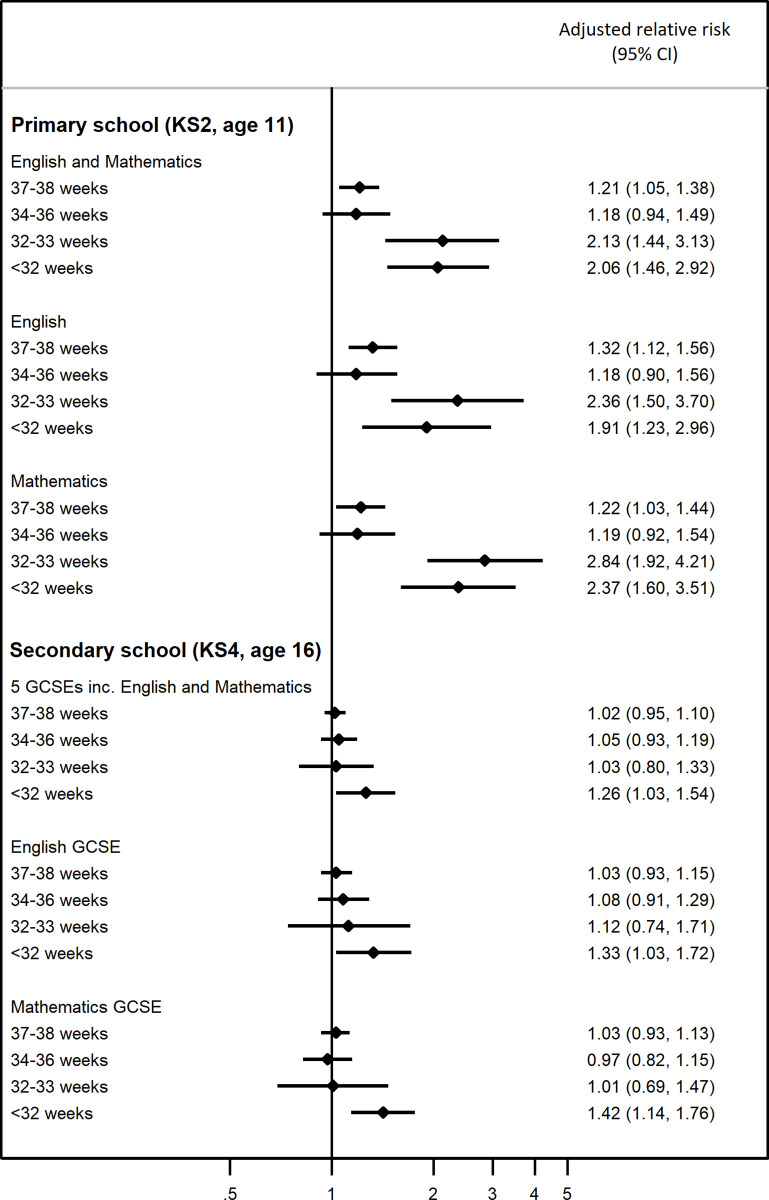
Adjusted relative risks for the association between gestational age at birth and attainment at Key Stage 2 (KS2; end of primary school, age 11) and at Key Stage 4 (KS4; end of secondary school, age 16) in the Millennium Cohort Study children born 2000–2001 in England. (GCSE: General Certificate of Secondary Education).

The proportion of pupils not achieving the expected level of attainment in both English and Mathematics at the end of primary school increased with earlier gestational age at birth from 16.1% in the full term group to 39.4% in the very preterm group (Table 4). Following adjustment for confounders, children born at early term gestations had an adjusted relative risk of 1.21 (95% CI 1.05, 1.38) (Fig 2). Children born late preterm (34–36 weeks) had a similarly elevated adjusted relative risk; however, this was not statistically significant (aRR = 1.18; 0.94, 1.49). Children born moderately and very preterm were at higher risk of not achieving the expected level of attainment at primary school: aRR = 2.13 (95% CI 1.44, 3.13) and aRR = 2.06 (95% CI 1.46, 2.92), respectively.

**Table 4:** The association between gestational age at birth and educational outcomes at the end of primary school (Key Stage 2; age 11) within the Millennium Cohort Study sample.

	Very preterm	Moderately preterm	Late preterm	Early term	Full term	Total
	<32 weeks	32–33 weeks	34–36 weeks	37–38 weeks	39–41 weeks	
**N**	74	79	416	1,458	4,923	6,950
**Did not achieve the expected level in both English & Mathematics**						
**n (%) ^1^**	27 (39.4)	25 (34.1)	86 (20.0)	279 (20.5)	757 (16.1)	1,174 (17.7)
**RR (95% CI)**	2.45 (1.77, 3.38)	2.12 (1.41, 3.18)	1.24 (0.97, 1.58)	1.27 (1.10, 1.47)	1	
**Adjusted RR (95% CI)**	2.06 (1.46, 2.92)	2.13 (1.44, 3.13)	1.18 (0.94, 1.49)	1.21 (1.05, 1.38)	1	
**Did not achieve the expected level in English**						
**n (%)**	17 (26.7)	18 (27.1)	60 (13.9)	206 (15.5)	495 (10.8)	796 (12.3)
**RR (95% CI)**	2.48 (1.63, 3.77)	2.52 (1.56, 4.06)	1.29 (0.95, 1.74)	1.44 (1.21, 1.72)	1	
**Adjusted RR (95% CI)**	1.91 (1.23, 2.96)	2.36 (1.50, 3.70)	1.18 (0.90, 1.56)	1.32 (1.12, 1.56)	1	
**Did not achieve the expected level in Mathematics**						
**n (%)**	23 (32.8)	24 (33.0)	64 (14.9)	211 (15.6)	581 (12.3)	903 (13.6)
**RR (95% CI)**	2.66 (1.81, 3.91)	2.68 (1.77, 4.06)	1.21 (0.93, 1.59)	1.26 (1.06 1.50)	1	
**Adjusted RR (95% CI)**	2.37 (1.60, 3.51)	2.84 (1.92, 4.21)	1.19 (0.92, 1.54)	1.22 (1.03, 1.44)	1	

^1^ percentages and means are weighted to account for study design, non-response and loss to follow-up by age seven

^2^ Adjusted for maternal education, maternal age, smoking during pregnancy, child sex, whether firstborn, whether multiple birth, month of birth

The pattern of risk found for not achieving the expected level of attainment in English and Mathematics separately was generally similar to that of the main outcome. In children born at early term and late preterm gestations, the adjusted relative risk for not achieving the expected level of attainment in English or Mathematics was approximately 1.2–1.3 (Table 4, Fig 2). In children born moderately and very preterm the adjusted relative risk was higher: 2.4 and 1.9 respectively for English and 2.8 and 2.4 respectively for Mathematics.

### Gestational age and academic attainment at the end of secondary school (KS4; age 16)

Almost half (45.2%) of the pupils in our sample did not pass five GCSEs including English and Mathematics. This proportion did not vary substantially by gestational age in those born at 32 weeks or above, but was much higher (60.3%) for pupils born very preterm (Table 5). After adjustment for confounders, pupils born very preterm had an aRR = 1.26 (95% CI 1.03, 1.54) for not achieving this outcome compared with pupils born at full term, while pupils born at later gestational ages were not found to have a risk that differed from those born at full term (Fig 2).

**Table 5:** The association between gestational age at birth and GCSE outcomes at the end of secondary school (Key Stage 4; age 16) within the Millennium Cohort Study sample.

	Very preterm	Moderately preterm	Late preterm	Early term	Full term	Total
	<32 weeks	32–33 weeks	34–36 weeks	37–38 weeks	39–41 weeks	
**N ^1^**	81	80	408	1,498	5,064	7,131
Did not achieve 5 GCSEs at grades A*-C including English & Mathematics						
**n (%) ^2^**	45 (60.3)	35 (46.1)	192 (47.9)	659 (46.8)	2,164 (44.2)	3,095 (45.2)
**RR (95% CI)**	1.36 (1.12, 1.67)	1.04 (0.78, 1.39)	1.08 (0.95, 1.24)	1.06 (0.98, 1.14)	1	
**Adjusted ^3^ RR (95% CI)**	1.26 (1.03, 1.54)	1.03 (0.80, 1.33)	1.05 (0.93, 1.19)	1.02 (0.95, 1.10)	1	
**Did not pass English**						
**n (%)**	29 (41.0)	21 (31.3)	118 (30.4)	400 (29.2)	1,281 (26.9)	1,849 (27.8)
**RR (95% CI)**	1.52 (1.14, 2.04)	1.16 (0.75, 1.80)	1.13 (0.94, 1.36)	1.09 (0.97, 1.12)	1	
**Adjusted RR (95% CI)**	1.33 (1.03, 1.72)	1.12 (0.74, 1.71)	1.08 (0.91, 1.29)	1.03 (0.93, 1.15)	1	
**Did not pass Mathematics**						
**n (%)**	37 (49.0)	24 (31.3)	131 (32.1)	467 (34.3)	1,575 (32.5)	2,234 (33.0)
**RR (95% CI)**	1.51 (1.20, 1.90)	0.97 (0.64, 1.45)	0.99 (0.83, 1.18)	1.05 (0.95 1.17)	1	
**Adjusted RR (95% CI)**	1.42 (1.14, 1.76)	1.01 (0.69, 1.47)	0.97 (0.82, 1.15)	1.03 (0.93, 1.13)	1	
**Attainment 8 score**						
**Mean (SD)**	39.2 (21.5)	44.8 (21.5)	44.7 (19.9)	44.9 (20.2)	45.9 (19.6)	45.5 (19.8)
**Mean difference (95% CI)**	-7.1 (-12.2, -1.9)	-1.0 (-7.6, 5.5)	-1.3 (-3.8, 1.2)	-1.1 (-2.5, 0.3)	0	
**Adjusted mean difference (95% CI)**	-5.4 (-10.2, -0.6)	-0.9 (-6.2, 4.4)	-0.7 (-3.0, 1.6)	-0.4 (-1.7, 0.9)	0	

^1^ Total sample size for the analyses of English, Mathematics and ‘Attainment 8’ were N = 7,127

^2^ percentages and means are weighted to account for study design, non-response and loss to follow-up at age seven

^3^ Adjusted for maternal education, maternal age, smoking during pregnancy, child sex, whether firstborn, whether multiple birth, month of birth

A similar pattern of results was found when examining English and Mathematics GCSEs separately. Overall, the proportion of all pupils not passing English was 27.8% and the proportion not passing Mathematics was 33.0% (Table 5). In adjusted analyses, the risk for not passing these subjects was not significantly higher in pupils born at 32 weeks or above compared with pupils born at full term. However, pupils born very preterm had an adjusted relative risk of 1.33 (95% CI 1.03, 1.72) for not passing English and 1.42 (95% CI 1.14, 1.76) for not passing Mathematics (Fig 2).

The mean ‘Attainment 8’ score for the pupils in our sample was 45.5. To put in context, a student who achieved the lowest pass in English, Mathematics and six additional subjects would have a score of about 40 [43]. The ‘Attainment 8’ score did not vary substantially by gestational age for those born at 32 weeks or above (Table 5). However, pupils born very preterm scored on average 7.1 (95% CI -12.2, -1.9) points lower than pupils born at full term. After adjustment for confounders the mean score difference was reduced to 5.4 (95% CI -10.2, -0.6). As an example, this is equivalent to achieving one grade lower in English, Mathematics and one other subject.

## Discussion

### Summary of findings

Overall, our findings suggest that birth at any gestational age earlier than full term (39–41 weeks), including birth at early term (37–38 weeks), is a risk factor for poor attainment at the end of primary school. The risk is more pronounced in children born before 34 weeks of gestation. However, by the time pupils reach the end of compulsory education, only those born very preterm (before 32 weeks of gestation) remain at risk of poorer attainment compared to pupils born at full term. Specifically, at the end of primary school, pupils born at early term gestations and those born late preterm (34–36 weeks) were found to have an approximately 20% higher risk of not achieving the expected level of attainment in both English and Mathematics compared with children born at full term. In contrast, pupils born moderately preterm (32–33 weeks) and very preterm (<32 weeks) were at approximately double the risk of not achieving the expected level of attainment in English and Mathematics compared with pupils born at full term. At the end of secondary school however, attainment was not associated with gestational age, except in the pupils born very preterm, assessed through two measures–not passing five GCSEs including English and Mathematics, and a continuous ‘Attainment 8’ score. Following adjustments, these pupils had a 26% elevated risk of not passing five GCSEs, and an average reduction of 5.4 points in the ‘Attainment 8’ score compared with pupils born full term.

### Strengths and limitations

Our study is based on a large sample of children born in England, which enables assessment of the entire spectrum of gestational age with follow-up extending until the end of compulsory education at age 16 years. We used routinely collected educational data to assess pupils’ attainment at the end of primary and secondary school based on standardized, curriculum-based, national attainment tests. A comparison between the level of attainment of the pupils included in our sample and those published in annual statistics for England indicates that the sample was generally representative of the population of children living in England. Furthermore, we were able to examine attainment in English and in Mathematics separately, which enabled us to assess the association between gestational age and learning in these two subjects.

An additional strength of our study is the use of two distinct measures for assessment of overall achievement in secondary school (five GCSEs including English and Mathematics and ‘Attainment 8’), which increases confidence in the findings. Furthermore, the ‘Attainment 8’ score is not limited to GCSE-approved academic subjects. Rather, in addition to English and Mathematics (double weighted), the ‘Attainment 8’ score can include a range of academic, technical and vocational qualifications.

The study’s main limitation is potential selection bias due to missing educational data for some pupils. Although the proportion of missing data was not very high overall (12.1% for primary and 9.7% for secondary school), it was higher in the moderately and very preterm groups. The main reasons for missing educational data are parental non-consent to linkage or unsuccessful linkage. A previous study carried out on the MCS found that non-consent to linkage did not differ by child’s health status, but was more likely for children with lower cognitive scores [44]. Since lower cognitive scores are thought to be on the causal pathway between earlier gestational age and poor school outcomes, this suggests that the associations found in our study might be biased towards the null. Unsuccessful linkage, another possible reason for missing educational data, might be attributed to attendance at an independent school. It is estimated that only 7% of pupils in England attend independent schools [45], many of which are included in the NPD. Yet, some independent schools do not follow the national curriculum or report results to the NPD. If children born in the earlier gestational age groups are overrepresented in these schools, then this missing data might also bias our results and is an important consideration when interpreting the results for secondary school, where effects are null or small. Additionally, the study is limited by possible residual confounding, for example, due to unavailable data such as the cause of preterm birth, maternal or fetal conditions, or provision of antenatal corticosteroids.

### Interpretation

Our findings could potentially be explained by catch-up in academic attainment among pupils born at 32 weeks of gestation or above, that is, moderately or late preterm, or at early term. Although a single sample using a longitudinal design and harmonized outcome is required to provide solid evidence of trajectories in academic attainment, the findings of our cross-sectional analyses imply that catch-up is likely the case. The MCS sub-samples used for each of the time-points analysed were almost identical, with only 6% of the children not participating in both samples, so this does not likely explain the different pattern of risk observed between attainment in primary and secondary school. Moreover, the outcome measure of five GCSEs including English and Mathematics used to assess attainment at secondary school was more difficult to achieve than the measure used to assess attainment in primary school, as indicated by the proportion of all children achieving each: 54.5% for secondary and 82.3% for primary school. Yet pupils born at early term, late preterm and moderately preterm gestation, who were on average less likely than pupils born full term to attain the expected level in primary school, were on average just as likely to attain the expected level in secondary school, despite it being a more difficult measure to achieve. Furthermore, the ‘Attainment 8’ score, being a continuous measure, is more sensitive than a binary one, and nevertheless showed no association with gestational age at secondary school, apart from in the very preterm group.

Very preterm children appear to be a high-risk group with persistent difficulties in terms of educational outcomes. An earlier longitudinal analysis of routine educational records of children born in the former Avon region in England in the early 1990s demonstrated narrowing of the attainment gap between all children born preterm and those born at term during the primary school years [14]. The study found some additional evidence from sub-group analyses to suggest that, for pupils born very preterm, the gain seen in attainment during primary school may be lost again over early secondary school, suggesting there may be a different trajectory in this higher risk group. Studies of pupils in educational systems outside of England also indicate that only very preterm pupils may be at persistently higher risk for poor educational outcomes. For example, a Danish study found that earlier gestational age is associated with higher risk for non-completion of required final examinations at age 15–17, but the grade point average of pupils in this study was lower only for pupils born very preterm [46], a finding in line with our own. Likewise, a study in California concluded that the excess risk for low attainment in pupils born moderately or late preterm had waned by year 9 (i.e., age 15) [12].

Although birth at very preterm gestations is a risk factor for lower attainment at the end of compulsory schooling, our findings suggest that the magnitude of risk associated with being born very preterm is smaller than the risk associated with a number of other risk factors. For example, the unadjusted average ‘Attainment 8’ score of the pupils born very preterm in our sample was 7 points lower than that of pupils born full term, while pupils eligible for free school meals achieve, on average, 13 points less in ‘Attainment 8’ score than pupils not eligible [36]. Still, children born very preterm are more likely to come from disadvantaged socioeconomic backgrounds, and may therefore experience both risk factors as an additive effect, as suggested in previous studies [47, 48].

Our study progresses the understanding of the relationship between gestational age and risk of poorer attainment in Mathematics and English. At the end of primary school, the effect of earlier gestational age at birth was slightly stronger for Mathematics compared with English (e.g., aRR = 2.4 vs. 1.9 in the very preterm), but confidence intervals overlapped. At the end of secondary school, the relative risk for not passing Mathematics GCSE was not different than for English. Previous studies have found relatively lower attainment in mathematics than in reading, typically in samples of extremely preterm children and on examiner-administered achievement tests [17, 21, 22, 49]. However, we did not find strong evidence to suggest that earlier gestational age at birth has a greater adverse effect on performance in national attainment tests in Mathematics compared with English.

Our findings imply that birth at earlier gestational ages is an independent risk factor for poorer performance in primary school. This is potentially because of the neurodevelopmental sequelae associated with preterm birth. Our findings further suggest that the educational attainment of those born very preterm is affected longer term and still observed at the end of compulsory education.

Using the MCS sample, we previously demonstrated an association between earlier gestational age at birth and special educational needs at age 11 [7]. It is possible that the additional support provided to pupils born preterm assists in closing or narrowing the attainment gap by the time these pupils finish their compulsory education. However, our study has relatively small numbers in the gestational age groups of less than 34 weeks. Further studies with a longitudinal design and adequate sample size are required to examine the trajectory of attainment of different gestational age groups and to assess whether any improvement is attributable to additional support given to preterm pupils with special educational needs.

## Conclusion

Our findings show that although preterm children born at 32 or more weeks of gestation have worse attainment during primary school than children born at full term, they have similar attainment at the end of secondary schooling. At this stage, 52–54% of pupils born 32–38 weeks of gestation pass at least five GCSEs including English and Mathematics, similar to 56% of pupils born full term (39–41 weeks) that achieve this outcome. Further research is required in order to better understand the trajectory of academic attainment in this group of pupils, but this finding may be reassuring for parents. Children born very preterm (before 32 weeks of gestation), however, remain at higher risk of poor school attainment at the end of compulsory education when only 40% attain five GCSEs including English and Mathematics. These children may benefit from screening for cognitive and learning difficulties prior to school entry to guide the provision of additional support from the outset of schooling.
